# A three-step approach to close refractory persistent macular holes: a releasing-closing-tapping approach

**DOI:** 10.1007/s00417-023-06197-9

**Published:** 2023-08-10

**Authors:** Tong Su, Shuai He, Peiyao Mao, Chufeng Gu, Chunren Meng, Chuandi Zhou, Jili Chen, Zhi Zheng, Qinghua Qiu

**Affiliations:** 1 Shandong Eye Hospital, Eye Institute of Shandong First Medical University, Eye Hospital of Shandong First Medical University, Jinan, China; 2 Department of Ophthalmology, Shanghai General Hospital, Shanghai Jiao Tong University School of Medicine, Shanghai, People’s Republic of China; 3 National Clinical Research Center for Eye Diseases; Shanghai Clinical Research Center for Eye Diseases; Shanghai Key Clinical Specialty; Shanghai Key Laboratory of Ocular Fundus Diseases; Shanghai Engineering Center for Visual Science and Photomedicine, Shanghai engineering center for precise diagnosis and treatment of eye diseases, Shanghai, China; 4 Shibei Hospital, Jing’an District, Shanghai, People’s Republic of China; 5 Department of Ophthalmology, Tong Ren Hospital, Shanghai Jiao Tong University School of Medicine, No. 1111 Xianxia Road, Changning District, Shanghai, People’s Republic of China

**Keywords:** Adhesion, Persistent macular hole, Reoperation, Vitrectomy

## Abstract

**Purpose:**

The aim of this study was to assess the efficacy and safety of a novel releasing-closing-tapping approach in the treatment of persistent macular holes (PMHs) after initial surgery with internal limiting membrane (ILM) peeling.

**Methods:**

We retrospectively analyzed patients with PMHs after initial surgery with ILM peeling who were treated with a novel releasing-closing-tapping approach. After repeated pars plana vitrectomy (PPV), the surgeon effectively released the adhesion between the edges and retinal pigment epithelium (RPE) by gently scraping the retinal neuroepithelium. Then, the hole was converted into a transverse slit, and the edges were gently tapped flat so that they attached to the RPE, and no space was left under the edges. Finally, air tamponade was carried out. The primary outcome measures included MH closure and the change in best-corrected visual acuity (BCVA) from preoperatively to postoperatively.

**Results:**

The study included 11 PMH patients with a mean age of 63.82 ± 3.31 years. The mean minimum linear diameter of PMHs was 666.3 ± 208.1 μm, and the mean basal diameter was 1547.2 ± 351.8 μm. MH closure was achieved in 90.9% (10/11) of eyes, with significant improvement of visual acuity from 1.19 ± 0.30 logMAR to 0.65 ± 0.29 logMAR postoperatively.

**Conclusion:**

The releasing-closing-tapping approach with repeated PPV is a simple, effective, and safe surgical procedure for refractory PMHs after initial surgery with ILM peeling that can significantly improve the visual outcome and achieve a high surgical success rate.

**Supplementary Information:**

The online version contains supplementary material available at 10.1007/s00417-023-06197-9.

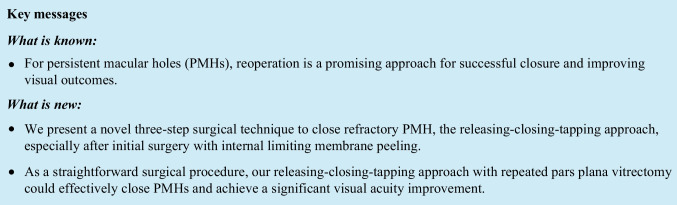



## Introduction

A full-thickness macular hole (MH) is a full-thickness defect involving the neurosensory retina at the anatomical fovea. Pars plana vitrectomy (PPV) with internal limiting membrane (ILM) peeling and gas tamponade has become the standard procedure for surgical treatment for MH since the first report by Kelly and Wen-del in 1991 [1]. In recent years, with the development of surgical treatments, such as extended ILM peeling and inverted ILM flaps, the anatomical closure rate has reached 90% [2–5]. Although these modified techniques have increased surgical success, some MHs fail to close after primary vitrectomy, which results in persistent MHs (PMHs). The incidence of PMH ranges from 8 to 44% [6] and has been reported to be positively correlated with the size and duration of the initial MH [7, 8].

Reoperation is reported to be a promising approach to achieve successful closure of PMHs and improve visual outcomes. Secondary attempts include repeated PPV with modified techniques, such as enlargement of the ILM rhexis [9], ILM translocation [10], lens capsule flap transplantation [11], autologous neurosensory retinal free flap transplantation [12], human amniotic membrane plug transplantation [13], induction of macular detachments with subretinal blebs [5], MH hydrodissection [14], and radial retinal incisions [15]. With these approaches, antero-posterior and tangential tractional forces are eliminated, MH stiffness is relieved, and a scaffold is implanted for glial proliferation to promote adhesion.

However, some MHs have been observed after secondary surgery; wherein, a foveal defect of the neurosensory retina persists. A possible reason is the adhesion between the MH edges and the underlying retinal pigment epithelium (RPE) [14, 16]. Despite partial success in MH closure, visual improvement is only modest with these techniques, and surgery remains complicated and challenging. Furthermore, few studies examining PMHs with a duration ≥ 6 months and aperture diameter ≥ 400 μm (refractory PMH) have been reported. Therefore, simple surgery options are still needed for PMH treatment.

We present a novel surgical technique consisting of repeated PPV combined with a three-step approach as follows: first, the adhesion between the MH edges and RPE is gently released to decrease the size of the hole; second, the edges of the hole are approximated; and finally, the edges are tapped flat to promote attachment of the edges to the RPE. We call this technique the releasing-closing-tapping approach. The aim of this study is to present the surgical technique and evaluate its efficacy and safety in the reoperation of patients with refractory PMHs.

## Materials and methods

### Patients

This retrospective consecutive case series study included 11 patients (11 eyes) with full-thickness MHs with a failed previous vitrectomy surgery, including failed MH closure with retinal detachment after silicone oil removal, who were then treated with this modified surgical technique. The patients were treated by a surgeon (Q. Q.) at Shanghai General Hospital, the affiliated hospital of Shanghai Jiao Tong University School of Medicine, from July 2020 to August 2021. All patients had previously undergone PPV with an area of ILM peeling more than 3 disk diameters and tamponade with air or silicone oil during their primary surgery.

The parameters recorded included the age, sex, ocular history, clinical manifestations, BCVA before reoperation and at each follow-up visit, MH appearance visualized with spectral-domain optical coherence tomography (OCT, Cirrus, Carl Zeiss, Dublin, USA) scans before initial surgery and reoperations and at each follow-up visit, and lens status before and after reoperation, length of follow-up, the occurrence of any postoperative complications, or MH recurrence.

The duration of the PMH was determined from the moment the patient first noticed significant visual loss or as documented on any available previous ophthalmologic examination report until the reoperation. The minimum hole width was measured at the narrowest point of the hole in the mid retina, and the basal diameter was measured at the largest hole point of the hole above the RPE.

The study was conducted in accordance with the Declaration of Helsinki and was approved by the Research Ethics Committee of Shanghai General Hospital. All patients were fully informed of the purpose, procedure and possible complications of the operation, and provided written informed consent.

The primary outcome measures included MH closure and the change in BCVA. MH closure was defined as complete sealing of the MH without bare RPE on OCT imaging. The foveal contour at a minimum 6-month follow-up was recorded based on its cross-sectional appearance on OCT. Examinations included anterior segment assessment, intraocular pressure, and clinical evaluation of the posterior segment. BCVA assessment and OCT were performed at every visit.

### Surgical technique

**Fig. 1:**
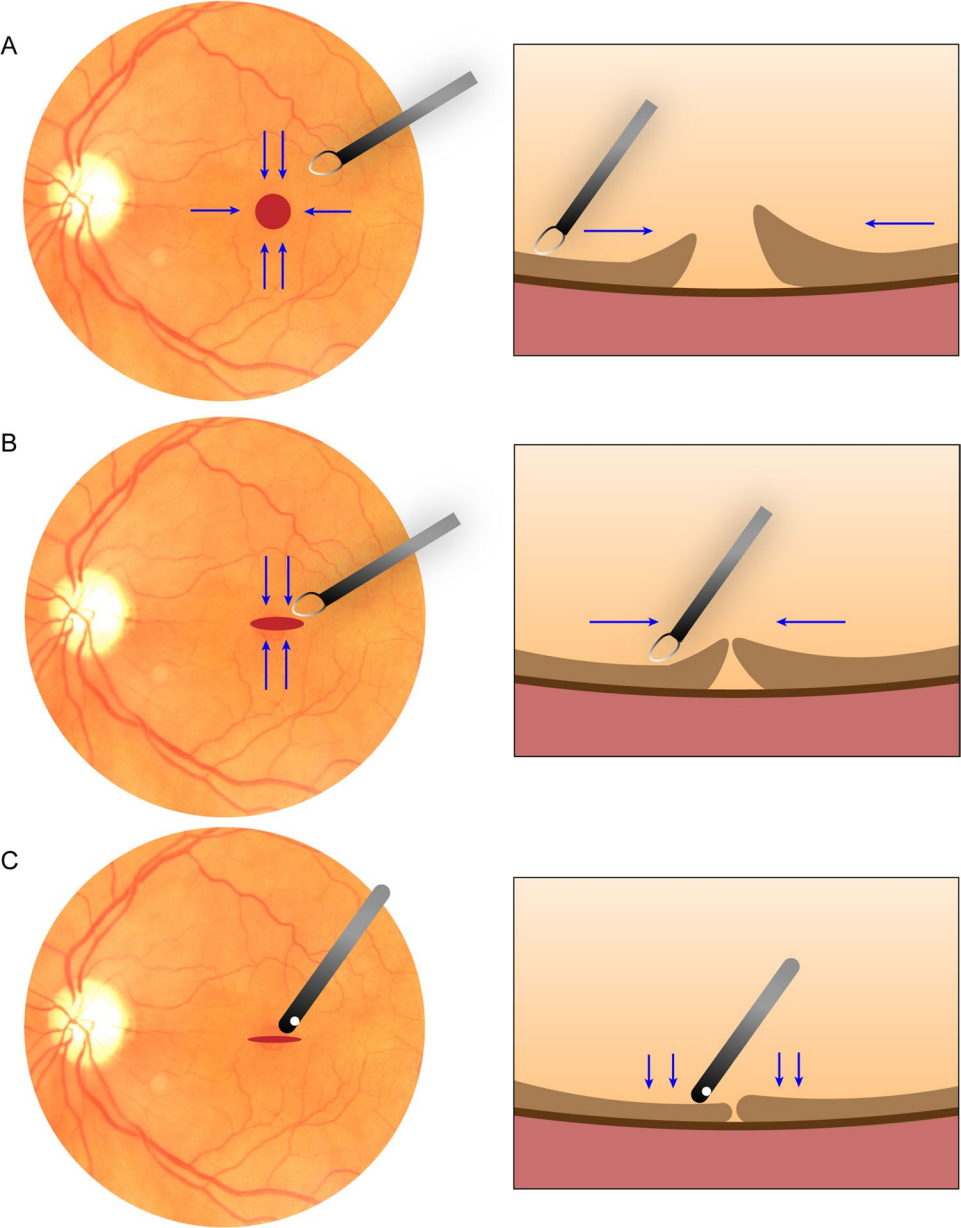
**A** A membrane loop was used to gently scrape the retinal neuroepithelium from approximately 2 or 3 disk diameters around the fovea to the center. **B** The edges of the hole were moved closer and were converted to a transverse slit with the membrane loop. **C** The blunt end a vitreous cutter was used to gently tap and flatten the edges, attaching them to the RPE and leaving no space under them

All 11 operations were performed under retrobulbar anesthesia using a 23-G vitrectomy system (Constellation®, Alcon, Fort Worth, TX, USA). After performing a standard three-port vitrectomy, the surgeon gently scraped the retinal surface from approximately 2 or 3 disk diameters around the fovea to the center with a membrane loop (FINESSE Flex loop; Alcon, Fort Worth, TX, USA), avoiding injury to the papillomacular bundle. In this process, a slight movement of the retinal neuroepithelium was observed, and the edges moved closer, which indicated release of the adhesion between the MH edges and RPE. Next, the surgeon focused on manipulating edges, approximating the edges of the hole to achieve reattachment of the fovea, and converting the round hole into a transverse slit with the membrane loop. Following this step, the edges were gently tapped flat with the blunt end of a vitreous cutter; thus, the retinal neuroepithelium of the edges attached to the RPE, and no space was left under the edges to prevent their movement after lifting the blunt end. If the edge was observed to be mobile as soon as the blunt end was lifted, the last step was repeated (Fig. 1; Supplementary video recording).

### Statistical analysis

Visual acuity measurements were transformed to logMAR values for statistical analysis. All continuous data are expressed as the mean ± standard deviation. All data were analyzed using SPSS 23.0 statistical software (SPSS Inc., Chicago, IL, USA). The Friedman test was used with *P* < 0.05 considered statistically significant.

## Results

The 11 patients included 3 males and 8 females. The baseline clinical characteristics are shown in Table 1. The mean age of the patients at the time of surgery was 63.82 ± 3.31 years (60–72 years). The duration of the MH ranged from 6 months to 4.5 years. The interval between the primary operation and reoperation ranged from 34 days to 53 months. Cataract extraction with intraocular lens implantation was previously performed on 10 eyes. All surgical procedures were successful. During the follow-up period, no intraoperative or postoperative complications occurred.

**Table 1:** Baseline characteristics of patients and their analyzed variables

Patient no.	Age(y)	Sex	BCVA preop. (logMAR)	BCVA 3 months postop. (logMAR)	BCVA postop. last visit (logMAR)	Lens status preop.	Lens status postop.	Initial MH minimum linear diameter (μm)	PMH minimum linear diameter (μm)	PMH basal diameter (μm)	MH status postop.	Follow-upperiod (month)	Interval time (day)	Duration of PMH (month)
1	60	F	0.7	0.52	0.4	Ps	Ps	387	562	2180	Close	12	37	6
2	63	F	1.3	1.3	1.3	Ps	Ps	497	1044	1405	Open	12	1612	53
3	66	F	1.22	1	0.82	Ps	Ps	937	768	1937	Close	12	142	10
4	72	M	1.1	0.7	0.52	Ps	Ps	836	717	1532	Close	12	126	8.5
5	62	F	0.82	0.7	0.7	Ps	Ps	493	417	1165	Close	14	56	7
6	61	F	1.85	1	0.4	Ph	Ps	560	906	1511	Close	19	251	12.5
7	61	F	1.3	1	0.85	Ps	Ps	605	563	1121	Close	9	373	16.5
8	64	M	1.15	1	0.4	Ps	Ps	509	404	1864	Close	9	34	6.5
9	63	M	1	1	0.4	Ps	Ps	No measure	466	1761	Close	9	385	17
10	65	F	1.3	0.52	0.52	Ps	Ps	792	831	1409	Close	6	58	6.5
11	65	F	1.3	1	0.82	Ps	Ps	859	652	1134	Close	6	48	6
Average (closed)	63.90 ± 3.48		1.18 ± 0.32	0.84 ± 0.21	0.58 ± 0.19			664.2 ± 194.5	628.6 ± 175.1	1561.4 ± 367.5		10.80 ± 3.91	151 ± 137.31	9.7 ± 4.3
Average (all)	63.82 ± 3.31		1.19 ± 0.30	0.89 ± 0.24	0.65 ± 0.29			647.5 ± 190.9	666.3 ± 208.1	1547.2 ± 351.8		10.91 ± 3.73	283.82 ± 459.37	13.6 ± 13.7

*PMH* persistent macular hole, *BCVA* best-corrected visual acuity, *F* female, *M* male, *Preop*. preoperative, *Postop*. postoperative, *Ps* pseudophakic, *Ph* phakic

After excluding the patient no. 9, who was diagnosed with macular rhegmatogenous retinal detachment and did not undergo measurement of the initial macular diameter, the mean initial minimum linear diameter was 647.5 ± 190.9 μm (387–937 μm). Although 6/11 MHs decreased after the initial surgery, all MHs were greater than 400 μm in diameter (large MH), with a PMH mean minimum linear diameter of 666.3 ± 208.1 μm (404–1,044 μm). The PMH basal diameter of the MHs was 1547.2 ± 351.8 μm (1121–2180 μm).

**Fig. 2:**
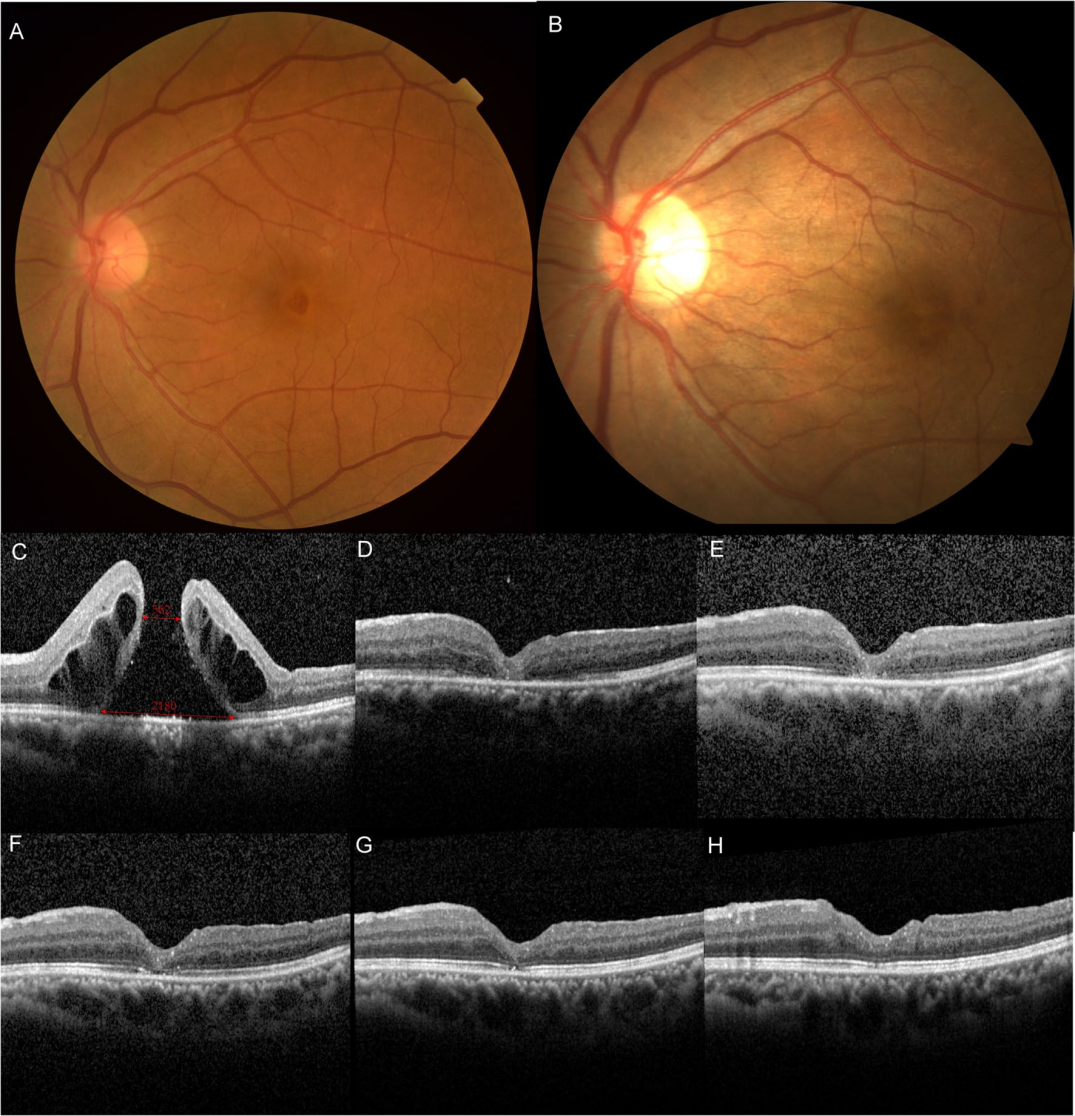
Preoperative fundus photograph and OCT of a PMH prior to reoperation (**A**, **C**). Postoperative fundus and OCT scans showing anatomical closure of the MH with gradual restoration of the ellipsoid zone 1 week (**B**, **D**), 1 month (**E**), 3 months (**F**), 6 months (**G**), and 1 year (**H**) after surgery

Anatomical MH closure was achieved, as determined by OCT, in 10 of the 11 patients (90.9%) at 5–14 days after surgery (Fig. 2). Only one hole failed to close (patient no. 2); this hole had the longest duration (53 months) and only underwent the first step of our novel surgical technique. Then, a three-step operation was done again adequately and completely performed on patient no. 2 6 months after the second operation, and the MH finally closed; therefore, the final MH closure rate was 100%.

The average BCVA of all PMHs improved significantly from logMAR 1.19 ± 0.30 to logMAR 0.65 ± 0.29 at the last follow-up (*P* < 0.001). The average BCVA improved to 0.89 ± 0.24 logMAR at 3 months follow-up but without statistical significance. After excluding the patient with an unclosed hole with the worst postoperative BCVA (1.3 logMAR), a statistically significant improvement in the BCVA of eyes with PMHs was revealed (*P* = 0.005).

## Discussion

The results of this retrospective study indicated that the novel reoperation approach was effective and safe for releasing adhesions between the MH edges and RPE to promote the attachment of the MH edges and thus successfully close refractory PMHs. Following this approach, MH closure was achieved in 90.9% (10/11) of eyes, with significant improvement of visual acuity after surgery and a final MH closure rate of 100%.

Adhesions of the retina to the RPE and retinal stiffness may contribute to PMHs [14, 16]. In our study, the minimum linear diameter of 80% (8/10) MHs (except patient no. 9, who exhibited retinal detachment) was greater than 400 μm before the initial surgery. The time from diagnosis to the first operation exceeded 1 month in 7 patients (63.6%), and their actual preoperative duration could have been longer. In addition to risk factors including large diameter, long duration, and long axial length, other factors that correlated with surgery failure included persistent vitreoretinal and epiretinal traction, possibly due to inadequate removal of the ILM or epiretinal membrane (ERM) regeneration [17, 18]. In our 11 cases, during the primary surgeries, all patients underwent enlargement of ILM peeling up to the vascular arcade or received an inverted ILM flap and air tamponade and maintained face-down positioning. We suggest that all original vitreous traction was eliminated during the first repair, but the MH failed to close [19]. However, the traditional technique for primary MH repair may not help to resist the traction forces generated by subretinal adhesions, thus leading to the first failed PPV.

Several simple options are available for secondary repairs, such as intravitreal injection of octafluoropropane (C3F8) and autologous blood or platelet-rich plasma application. Ying-Yi Chen et al. described the success rate in PMH to be 63% (12/19) only by early intravitreal injection of C3F8, but found MHs with original minimal diameter > 666 μm or persistent minimal diameter > 371 μm failed to close [20]. One of our patients (patient no. 2) underwent intravitreal injections of C3F8 with/without fluid/gas exchange more than once in another research-oriented hospital located in Beijing after its initial surgery, but the hole still did not close. A larger study by Valentin Degenhardt et al. reported the final closure rate after re-vitrectomy with autologous platelet concentrate was 60.2% (62 of 103 eyes) [21]. Although the probable reason why the closure rate of our study is that the sample size in our study was too small, the maximum diameter of their closed group was 563 μm, smaller than the mean minimum linear diameter (628.6 ± 175.1μm) of our closed PMH. Therefore, we can conclude that our surgical procedure may be more beneficial for large diameter PMH compared to these easier options.

Induction of macular detachments with subretinal blebs and MH hydrodissection or retinal massage was reported to effectively reduce the stiffness of the retina and subretinal adhesions. Roger Wong first massaged the retina radially using a tano silicone-tipped scraper to approximate the edges after performing puncture retinotomies and infusing balanced salt solution to produce a localized retinal detachment with a 41-G needle [22]. However, he only reported successful closure of three MHs with a thinned retina at the fovea by postoperative OCT. Rubin et al. also performed gentle centripetal massage using a backflush cannula to achieve perifoveal centripetal macular displacement after subretinal fluid application [23]. They reported complete anatomical closure in 6 of 7 (85.7%) large traumatic MHs. In contrast, there have been few reports of surgical options for PMHs, especially refractory PMHs with large diameters and long durations.

Application of this surgical approach—combining subretinal fluid and massage—to recurrent or PMHs was reported to be effective by Osman Abdelzaher Mohammed et al. [24]. After creating a neurosensory blister and massaging toward the center with a diamond-dusted scraper, they used end gripping forceps to pinch the temporal edges of the MH. In their study, four recurrent MHs underwent type 1 closure. Although all of these previous reports demonstrated the reliability and efficacy of this surgical approach, the procedure of infusing subretinal fluid, which promotes neuroretinal displacement and localized retinal detachment close to the fovea, is complicated and difficult to perform. Additionally, a learning curve is required to carefully control the procedure to prevent further trauma to the macula and creation of a large area of retinal detachment.

The retinal massage technique alone, without subretinal fluid application, has been previously reported for MH apposition. Chakraborty et al. [25] recently described retinal massage under air in a centripetal direction, i.e., using a 27-G soft silicone tip, after vitrectomy and ILM peeling for 41 eyes with a minimum diameter of 550 μm. The authors reported a closure rate of 100%, which was similar to our final closure rate, but did not consider whether this technique has a similar protective effect in PMHs. Furthermore, the mean basal diameter of our PMHs (1547 ± 352 μm) was significantly larger than that of Chakraborty’s study (835 ± 208 μm). The 27-G soft silicone tip that Chakraborty used to perform retinal massage and the fluid gas exchange and backflush cannula used by Rubin et al. may generate negative pressure during the operation, which could cause damage when it closely opposes the surface of the retina. Instead of the instruments described above and the tano silicone-tipped scraper used by Roger Wong, we chose the membrane flex loop, which used the smallest amount of power to slightly shift the largest area of retina. Additionally, this technique allowed the surgeon to more easily perform centripetal massage. The defects of the ellipsoid zone and ELM gradually healed and become continuous 6 months postoperatively in all cases, achieving successful closure.

Every step of our novel approach was convenient and necessary for the successful closure of PMHs after ILM peeling. The releasing step was similar but not identical to the retinal massage reported by others because the key point was slight movement of the retinal neuroepithelium, which in our opinion involved not only mechanical pulling but also shifting of the perifoveal retina through the membrane loop. In contrast to MHs that are closed after the first surgery, the mobility of the retina edges of the refractory PMHs with a duration of more than 6 months (54.5%, 6/11 cases) was not sufficient for the edges to connect to each other with wider ILM peeling, adjustment of the ILM flap with gas tamponade and face-down positioning. Therefore, range-expanding perifoveal retina release was necessary, especially for PMHs with large diameters. During the first three-step procedure in patient no. 2, the hole was not converted into a transverse slit and was not gently pressed flat after the edges moved closer after undergoing the release step, and the PMH has a long duration (4.5 years) and large diameter (minimum diameter 1044 μm, maximum basal diameter 1405 μm), which likely together contributed to treatment failure. After adequately and completely performing the three-step procedure in the third surgery for patient no. 2, the edges connected to each other and the RPE underneath, resulting in final closure.

The trauma produced by ILM peeling has been proposed to promote glial cell proliferation, including Müller cells, which promotes MH healing [26]. However, all 11 eyes underwent ILM peeling during the first operation. We hypothesized that after the edges moved closer, massaging the holes into a transverse slit that followed the course of the optic nerve fiber and flattening the edges by tapping to attach them to the RPE provided a scaffold for glial cell proliferation. Studies have reported that the maintenance of foveal hyperreflective lesions at long follow-up time is associated with worse visual recovery [27]. According to Wakabayashi and associates, the proliferating glial cells fill the foveal defect before bridging of the reapproximated ELM occurs, and subsequent reestablishment of the normal tomographic external retina profile at the central fovea is impeded, resulting in failure to recover the hyperreflective line corresponding to the ELM [28]. In the third step, slight tapping to remove the space under the edges avoided their movement after lifting the blunt end of the vitreous cutter, which could also promote bridging of the ELM to some extent.

However, caution should be exercised when interpreting the findings of this study because of a number of limitations, including the retrospective design, small sample size recruited from a single tertiary institution, and short follow-up periods (6–12 months). Our results might not be generalizable to the entire PMH population and cannot be used to determine the long-term prognosis for the treatment. In addition, all surgeries were performed by a single surgeon; therefore, selection bias may exist. Retinal nerve fiber layer OCT and microperimetry were not performed. Therefore, large, multicenter, randomized controlled trials are needed in the future to determine the clinical value of retinal massage in reoperation for PMHs.

In this study, we presented a novel reoperation treatment option for refractory PMHs after initial surgery with ILM peeling, and our surgical results confirmed its easy and fast application, with minimal adverse events and a high surgical success rate. Further prospective randomized case control studies with a larger sample size should be conducted to evaluate the clinical results and safety of this technique.

### Supplementary Information

ESM 1 Supplementary video 1.mp4 (MP4 320589 kb)

## Data Availability

All data associated with the present paper are included in the manuscript.
